# Assessing WHO’s influence: A randomized conjoint experiment on vaccine endorsements in diversified global health systems

**DOI:** 10.1371/journal.pgph.0005410

**Published:** 2025-11-21

**Authors:** Naoko Matsumura, Renu Singh, Christopher Howell, Tobias Heinrich, Matthew Motta, Yoshiharu Kobayashi

**Affiliations:** 1 Graduate School of Law, Kobe University, Kobe, Hyogo, Japan; 2 School of Government and International Affairs, Durham University, Durham, United Kingdom; 3 Political Science Department, College of Charleston, Charleston, South Carolina, United States of America; 4 Department of Political Science, The University of Houston, Houston, Texas, United States of America; 5 School of Public Health, Boston University, Boston, Massachusetts, United States of America; 6 School of Politics and International Studies, University of Leeds, Leeds, United Kingdom; PLOS: Public Library of Science, UNITED STATES OF AMERICA

## Abstract

During a novel pandemic, significant uncertainty drives individuals to seek expert guidance on preventive measures such as vaccination. Yet, it remains poorly understood how people process information in a highly complex landscape of global health governance where multiple experts may offer competing, repetitive, or contradictory advice. This study investigates the influence of World Health Organization (WHO)’s endorsements of vaccines amidst this environment. In fall 2020, we conducted a randomized conjoint experiment in Canada (832 respondents, 8,320 profiles evaluated), Japan (1,474, 14,740), and the United States (1,001, 10010), focusing on both whether and when people choose to vaccinate against COVID-19. Our experiment randomly varied exposure to vaccine endorsement information from several prominent global health governance players, including the WHO, the Centers for Disease Control and Prevention (CDC), Oxford University, and the Gates Foundation; and, unlike previous studies, different combinations of these endorsements were used. WHO endorsements increase individuals’ willingness to vaccinate more quickly, even when accompanied by endorsements from other credible organizations. However, the effect of WHO endorsements is not significantly stronger than that of other organizations. Notably, the impact of the WHO’s endorsement diminishes as the number of endorsements from other organizations increases. The WHO has the greatest impact when it is the first (or among the first) of many organizations to endorse a vaccine as safe and effective, and it may help inspire public confidence in less effective (but potentially lifesaving) vaccines. Overall, our study shows that WHO endorsements significantly reduce vaccine hesitancy, but endorsements from other global actors can exert comparable effects. This highlights that effective global health communication thus depends not on a single authoritative voice but on the timely coordination of multiple credible actors, underscoring the resilience of the global health system in promoting vaccine acceptance.

## Introduction

During a novel pandemic, uncertainty about the pathogen and the effectiveness of medical and policy responses drives individuals to seek guidance from health experts on preventive measures like vaccination [1,2]. In particular, the complexity of understanding how immunity and vaccines work poses a significant challenge for the general public [3,4]. In turn, many people look to experts and their endorsements and recommendations for guidance on what to do to minimize harm [5,6]. When it comes to vaccines, one of the most important tools to protect against harm from a pathogen, previous research shows that people act upon endorsements from physicians [7,8], public health experts [9], national health institutions [1,10,11], and international organizations (IOs) [10,12–14].

It remains poorly understood how individuals navigate the complex information environment created by simultaneous guidance from multiple experts. The complexity of cue-taking significantly increases when messages from various sources are competing, repetitive, or contradictory [15–17]. This challenge is particularly relevant in global health governance, where the number and diversity of actors involved has increased. While the World Health Organization (WHO) remains central to providing guidelines and recommendations, its dual identity, as both a technical, evidence-based body and a political organization representing diverse member states [18], may influence perceptions of its credibility as a cue source [19]. Indeed, alongside the WHO, many other state and non-state entities, such as foundations, universities, and national health agencies, equipped with substantial financial resources, expertise, and public rapport, actively offer their own health guidance today [20–23]. Understanding how the active involvement of these diverse actors shapes the public’s response to health guidance remains an open and critical question.

While existing work mentioned above has examined the effect of endorsements from various experts, their research presented endorsements exclusively *in isolation*. For example, Kreps *et al*. and Motta conducted conjoint experiments in which participants are presented with two hypothetical vaccine profiles, each endorsed by different experts—e.g., one endorsed by the WHO and the other by the Centers for Disease Control and Prevention (CDC)—along with other attributes, such as side effects and efficacy levels [10,13]. Such design enables us to examine whether a *particular* expert’s endorsement boosts public vaccine uptake relatively to any other, if only one were to endorse the vaccine. However, the design prevents us from assessing the influence of an endorsement when multiple other voices endorse (or do not endorse) the same vaccine simultaneously, a scenario that is clearly present in the diversified multi-actor global health system today.

In this study, we advance our understanding of vaccination intentions by designing and conducting an experiment that allows for multiple concurrent expert endorsements of vaccines. We focus on the role of WHO’s vaccine endorsements in the presence of other expert endorsements as it is the most prominent IO in global health governance. The other potential endorsers are key players in global health governance, including the CDC, Oxford University, and the Gates Foundation, whose endorsements can compete with, diminish, or complement the credibility and influence of WHO recommendations.

Our experimental design offers several advantages over previous approaches. First, by presenting only one endorser and endorsement per vaccine, prior studies may have amplified the influence of the endorsement by the WHO and others. In contrast, our approach more closely reflects real-world complexities, potentially improving the external validity of our findings by identifying effects relevant in such complex information environments. Of course, it is possible that people might overlook endorsements from other actors, but whether this occurs remains an empirical question, which our design allows us to answer.

Second, our experiment is also designed not only to assess endorsements but also non-endorsements from key experts. This approach allows us to capture the potential divergence in expert opinions. For example, we can compare how the public perceives a vaccine that is exclusively recommended by the WHO versus one that the WHO recommends but experts in the CDC do not endorse. Such contextual differences could significantly influence public perception.

Third, our design also lets us examine the extent to which the WHO plays a unique role in global health governance. Due to its prominence and visibility in elite discourse and media, we may suspect that the WHO has a unique ability to influence public opinion. However, the role of WHO endorsement may be substitutable—i.e., its influence may diminish when other experts simultaneously give similar guidance and endorsements. Alternatively, however, it is also theoretically possible that endorsements from other experts could complement and increase the influence of WHO endorsements. Our experiment can be used to address such important questions.

Finally, our experiment incorporates an innovative design choice from Kobayashi *et al*., emphasizing the crucial aspect of *when* respondents intend to get vaccinated [24]. Understanding the timing of vaccine uptake is important for effective rollout, as public hesitancy to get vaccinated early can significantly slow this process. By incorporating this design, we simulate how the share of vaccinated individuals (measured by their intent) evolves throughout the course of a pandemic. This simulation provides valuable insights that are directly relevant to public health strategies.

We conducted this experiment during Fall 2020 in Canada (832 respondents, 8,320 vaccine profiles evaluated), Japan (1,474, 14,740), and the United States (1,001, 10,010), focusing on not only whether or not people choose to vaccinate against COVID-19, but *how quickly* they might choose to do so. Several key findings emerge from our experiment. First, WHO endorsements increase individuals’ willingness to get vaccinated more quickly despite the presence of other credible endorsers. This finding is particularly noteworthy because, unlike existing evidence, it indicates that people value WHO endorsements despite having alternative expert opinions available. This suggests that concerns regarding the WHO’s alleged politicization and bias [19,25] might have been overstated, as the endorsements still positively affected people’s willingness to follow its recommendations across different countries.

Second, the influence of WHO endorsements on public opinion is not markedly greater than that of other organizations in the experiment. Across the three countries, the effects of WHO endorsements are similar in magnitude to the those of the CDC, Oxford University, and the Gates Foundation. This suggests that the WHO’s role in shaping public opinion during health crises is not unique compared to other key players in global health governance.

Third, the impact of WHO endorsements decreases as additional endorsements from other reputable global health actors emerge. This further supports the point that the WHO’s role is not uniquely critical, as other actors can effectively match and substitute its influence. Encouragingly, this highlights the resilience of a diverse, multi-actor system in global health governance. If others refrain from endorsing a vaccine, perhaps due to domestic politics, a WHO endorsement can significantly bolster vaccine uptake. Conversely, if the WHO were to be hamstrung, other endorsers can mitigate its absence. Thus, any credible endorsement can help increase uptake, with the first few endorsements being the most crucial.

Finally, while highly effective vaccines became available during the COVID-19 pandemic, future pandemics might not produce vaccines with similar efficacy. Highly effective vaccines typically require fewer endorsements, as their strong efficacy and safety profiles inherently appeal to the public. However, vaccines with lower efficacy or safety remain valuable when pathogens are highly dangerous. In such cases, endorsements become crucial, as clinical results alone may not sufficiently reassure the public. We find WHO endorsements can indeed matter for these lower-quality vaccine profiles, although this influence is context-specific.

In the next section, we outline the empirical and theoretical motivations underpinning our study and introduce the research questions this paper addresses. Then, we introduce our samples and experimental design. In the fourth section, we will present the results and our simulation exercise to study the impact of WHO endorsements for low- and high-quality vaccines. We end with a discussion of the policy implications and limitations of our study.

## Expert advice from multiple actors in global health

The WHO is the most important global health institution, staffed by medical experts recognized internationally for their technical expertise. Therefore, the guidance provided by the WHO is likely viewed as highly credible and shapes public perceptions and influencing health-related behaviors [5,6,26,27]. Indeed, the WHO maintains substantial cross-national trust, particularly evident during the COVID-19 pandemic [28]. In addition, trust in scientific authorities, including the WHO, positively correlates with increased public willingness to engage in recommended health practices, such as COVID-19 vaccination and compliance with preventive measures [29,30], suggesting the WHO’s pivotal role in disseminating authoritative scientific information during global health crises.

Despite its technical credibility, the WHO is inherently political, comprising member states with diverse political agendas and bureaucratic interests. This inherently political character may limit its effectiveness in shaping public opinion and behaviors [31]. When expert recommendations are perceived as politically motivated, their persuasiveness can diminish among the public [19] although evidence on this remains mixed [29,32]. Similar to experiences in previous pandemics, the WHO faced such credibility challenges during the COVID-19 pandemic, including allegations of political bias and bureaucratic inefficiency. These criticisms potentially undermined public trust in the WHO’s recommendations and guidance during this critical period [18,25,33,34].

While the WHO occupies a central position, it is only one entity within an increasingly complex landscape of global health governance [21]. This governance system includes diverse actors, ranging from national and local health authorities to non-governmental organizations (NGOs), foundations, private businesses, and academic institutions, each communicating expert guidance to the public. endorsements from diverse actors, such as the CDC, healthcare professionals, and political leaders, positively influence public health behaviors [1,7–11]. However, existing studies assess the effects of endorsements from these actors individually and in isolation, leaving unclear whether their influence remains robust or diminishes when multiple endorsements coexist within the same informational context.

This observation raises important questions about the comparative influence of the WHO versus other global health actors. Given its prominence, the WHO might remain uniquely influential. Conversely, other organizations, potentially perceived as equally competent and less politically motivated, could exercise comparable or even greater persuasive power. If true, this suggests the complexity and diversity within global health governance may create resilience by having multiple credible actors to effectively guide public behavior during health crises.

Furthermore, the combined effects of multiple endorsements remain an open empirical question. On one hand, endorsements might function as substitutes. That is, individuals may only need to hear the recommendation from a single actor they view as a credible source, after which they will tune out any additional information. Alternatively, endorsements might be complementary, with each additional endorsement that people encounter from a credible source further increasing their likelihood of accepting important recommendations on issues like vaccination. If endorsements reinforce one another, coherent and unified messaging across the governance landscape becomes critically important for effectively guiding public responses to global health threats.

## Materials and methods

We use an experiment to study the effect of expert endorsements on people’s vaccination intentions. Experiments are particularly advantageous for our research because they enable us to have control over the features of the vaccine, expanding upon the few actual vaccines available at the time of the survey (late 2020). While our experiment focuses on a participant’s self-reported vaccination intention, there is substantial evidence showing a strong link between self-reported intention and actual vaccine update [35–37].

### Subject recruitment

We conducted our survey in three counties: Canada, Japan, and the United States. Our multi-country, cross-national approach is designed primarily to assess the robustness and generalizability of our findings rather than to examine country-specific variations. Given this objective, we chose countries with varying attitudes toward the WHO, vaccine hesitancy levels, political relations with China, and COVID-19 infection rates at that time (in addition to practical considerations, including contextual familiarity, availability of high-quality survey participants, and financial constraints).

We recruited the respondents in late November 2020, after the first viable and effective vaccines were announced but before any were officially approved by a government. We recruited participants using Prolific, an online platform based in the United Kingdom where we set criteria and eligible individuals have the opportunity to participate if they meet criteria. Research demonstrates that Prolific recruits a more diverse and higher-quality participant pool than comparable online labor platforms (e.g., Amazon Mechanical Turk) and conventional online panels (e.g., Qualtrics) [40–42]. Specifically, we recruited 832 respondents from Canada (11/20/2020–11/24/2020) and 1,001 in the United States (11/23/2020–11/24/2020). For Japan, we used Yahoo! Crowdsourcing, a Japan-based online platform, to recruit 1,474 respondents (11/21/2020). These sample sizes are comparable to or exceed those commonly employed in standard conjoint experiments.

Unsurprisingly, our respondents were younger and more left/liberal-leaning and less right/conservative-leaning (see S1 Fig for the standardized differences in means between the sample and target population). While previous validation efforts show experiments using opt-in samples, such as from Prolific, consistently replicate the signs of treatment effect estimates carried out in random samples of the target populations [43,44], reweighting to match demographic moments of opt-in samples to the target populations’ can help bring magnitudes of the treatment effects more in line [45]. We turn to entropy balancing [46] to balance the country-specific samples so that the mean age and proportions of left/liberals, right/conservatives, and women match between the samples and the target datasets. Our reweighting procedure improves the overall balance of the data, though some imbalances remain, particularly with respect to education and gender in the Canadian and US samples (see S1 Fig). All the analyses below use these country-specific weights.

### Experimental design

We use a conjoint design, an approach previously used to study vaccine preferences [10,13]. In our study, we ask respondents to evaluate a series of five pairs of hypothetical vaccines, each differing in specific attributes. All attributes and potential realizations are summarized in Table 1. All realizations were drawn from a uniform distribution, recognizing ignorance about what a future vaccine might be like.

**Table 1:** Vaccine attributes and endorsements, possible realizations.

Attribute	Potential realizations
*Risk of severe side effects*	1 in 10,000
	1 in 1,000,000
*Risk of mild side effects*	1 in 10
	1 in 30
*Efficacy against severe symptoms*	50%
	70%
	90%
*Protection duration*	1 year
	5 years
*Vaccine origin*	China
	Germany
	United Kingdom
	United States
*Endorsed by CDC?*	Yes
	No
*Endorsed by Gates Foundation?*	Yes
	No
*Endorsed by World Health Organization?*	Yes
	No
*Endorsed by Oxford University?*	Yes
	No

Notes: All potential realizations for an attribute were equally likely.

We divide the attributes into two blocks: vaccine features and endorsements. The vaccine features include duration of effectiveness, risks associated with mild and severe side effects, efficacy against severe cases, and the vaccine’s country of origin—China, Germany, the United Kingdom, or the United States. These attributes enable us both to replicate and validate our conjoint design, and to examine whether endorsement effects hold for vaccines of lower overall quality.

Our objective is to assess how the persuasive impact of WHO endorsements compares with endorsements issued by other influential global actors. Building on scholarship on cue-taking [6,29,30], we theorize that individuals evaluate vaccine endorsements not only in terms of technical expertise but also with regard to perceptions of neutrality and independence. Endorsements from institutions seen as neutral and technically competent may therefore be more influential than those viewed as politically embedded or contested. This logic is particularly relevant for the WHO, which holds a dual identity. It is widely recognized as scientific authority, yet simultaneously an intergovernmental body whose decisions are vulnerable to politicization, a dynamic that became especially salient during the COVID-19 pandemic [18].

Guided by this framework, we selected three comparator organizations that (1) were salient across Canada, Japan, and the United States in late 2020, (2) exemplify distinct institutional bases of legitimacy, and (3) plausibly compete with or complement WHO endorsements. Oxford University embodies academic independence, with its credibility further reinforced by its prominent role in COVID-19 vaccine development. The Gates Foundation symbolizes global philanthropy and policy entrepreneurship, reflecting authority rooted in resources and prominence in global health advocacy. The CDC represents national bureaucratic authority with long-standing technical competence in public health, though its proximity to domestic politics may complicate perception of independence. Together with the WHO, these organizations span the major non-commercial sources of health guidance available to global publics during the pandemic. Their contrasts also allow us to investigate whether WHO’s influence is reinforced, substituted, or attenuated when endorsements come from institutions grounded in different forms of legitimacy.

We restrict the design to this set of four endorsers in order to maintain cognitive tractability for respondents, while still enabling systematic variation in the number of co-endorsements. This structure allows us to evaluate whether additional signals produce additive, complementary, or diminishing effects on the persuasive impact of WHO’s endorsement.

Each vaccine profile in our study indicates whether it is endorsed by these actors, reflecting their diverse influences and potential impacts. We use a checkmark symbol for endorsement and a dash sign for non-endorsement. The latter is expected to be understood as “no information” about the vaccine from that entity, rather than as suggesting disapproval. However, the absence of endorsement can be informative in itself as it might raise questions about the quality of the vaccine.

Prior to the profiles of vaccines, we provided respondents with brief introductions to the four endorsers and asked for them to rate their feelings towards these entities using a 100-point scale thermometer before introducing the hypothetical vaccines (see S1 File for greater details). As part of our original experimental design, we also included a specific treatment in the WHO introduction to assess if perceptions of bias affected its endorsement impact. We created two versions of the WHO introduction. Half of the respondents read that “the WHO is the world’s most important international organization dedicated to all matters of global health,” whereas the other half see the same introduction with an additional note saying “[during] the early days of the current Coronavirus pandemic, the WHO was sharply criticized for its deference to China where the novel coronavirus originated.”

We decided to omit this aspect of the analysis as our manipulation check—the thermometer scores—revealed that this additional note on China did not significantly alter perceptions of the WHO in two of the three countries surveyed, contrary to our intentions; in one country, the effect magnitude was tiny. Therefore, in the subsequent analyses, we will not distinguish between the two versions of the WHO and pool all responses. We elaborate on this decision in S1 Appendix.

### Outcome variable

Following the approach used by Kobayashi *et al*., we ask respondents to rate each vaccine in each pair using an ordinal scale to determine the expected timeframe for vaccine uptake [24]. This approach differs from the binary choice used in much previous research, offering two main advantages. First, our measure yields a richer set of data that goes beyond a simple yes/no binary. Second, the timing of vaccinations is crucial for public health outcomes, as achieving herd immunity necessitates widespread vaccination. Therefore, understanding the distribution of intended vaccination timings provides more insightful analysis as our simulations will show.

Respondents were given five options to express whether and when they would choose to get vaccinated if a vaccine were available at no cost. These options were: “Yes, within a month," “Yes, within 2-3 months,” “Yes, within 4-12 months,” “Yes, after a year,” and “No, never.” To simplify our analysis and the interpretation of results, we consolidated these responses into a three-level ordered variable: the “early” category for intentions within three months, “middle” for the 4-12 month timeframe, and “late" for wait times of a year or more, including “never.” This three-level ordinal variable offers a more detailed understanding of vaccination intentions. For the simulations of the vaccination shares, we will use all levels.

### Additional variables

Early in the survey, we ask respondents to answer a series of questions following the general vaccine hesitancy literature [38], including how they feel about vaccine safety and necessity [39]. We asked about their degree of agreement or disagreement with the statements (seven levels), “Adults should get all recommended vaccines", “Children should at all recommend vaccines", and “General speaking, recommended vaccines are safe." For each survey country, we take the three answers, treat them as approximately linear, standardize each by its mean and standard deviation, and then take the average. This is our index of vaccine attitudes. For the U.S. and Canada samples, Cronbach’s *α* rounds to 0.97 for each; in Japan, we dropped the third item, which improves the *α* from 0.59 to 0.83. We used simple imputation to fill missing vaccine attitudes index observations for 0.8% of respondents (n=27). Further, we also collect standard demographic information, such as age, gender, education, and ideological orientation. Since these differ slightly by country (see below), we relegate details to S1 File.

### Ethics statement

Research ethics approval for the experiment was obtained from the Research Ethics Committee of Graduate School of Law at Kobe University (020006) and the Office of Research Compliance at the University of South Carolina (Pro00105857) before it was run. The survey was conducted in Canada from 20/11/2020 to 24/11/2020, in Japan on 21/11/2020, and in the United States from 23/11/2020 to 24/11/2020. On the first page of the online survey (on Qualtrics), we provided respondents with all the necessary information about our study to ensure informed consent. This includes the researchers’ names, affiliations, and contact information; the purpose of the survey; a general explanation of what participation entails; benefits to participants (e.g., compensation); and the anonymous and voluntary nature of the survey. After reviewing this information, respondents were asked to confirm their informed consent. Additionally, there was no use of deception or misrepresentation.

## Results

We begin by detailing our approach and findings from the validation exercise of our conjoint design. Following this, we discuss the results about vaccine endorsements by the WHO in various contexts.

### Validating the conjoint design

While the general design of conjoint experiment has been validated to “work” [47], incorporating attributes from Kreps *et al*.’s work into our experiment helps evaluate participant engagement with our specific conjoint presentations [10]. Recovering experimental effects documented in previous work is particularly important for our study since our survey includes Canada and Japan in addition to the United States, two countries (to our knowledge) not used in conjoint designs on this subject at the time, and because we measure vaccination intentions in a distinct manner from previous conjoint-based work. Table 2 gives the results from ordered-probit models with errors clustered by respondent. In our dataset, each row represents a vaccine evaluated by a respondent, with the ordinal outcome being the speed at which they would choose to take the vaccine. The table omits the coefficient on demographic variables, which can be found in S2 Table.

**Table 2:** Estimates for vaccine uptake models.

	Canada	Japan	USA
Protection duration, 5 years	–0.05	–0.08	–0.07
	[–0.12; 0.02]	[–0.15; –0.01]	[–0.12; –0.02]
Efficacy, 50%	0.17	0.22	0.23
	[0.05; 0.30]	[0.14; 0.29]	[0.15; 0.31]
Efficacy, 90%	–0.30	–0.07	–0.31
	[–0.39; –0.20]	[–0.15; 0.00]	[–0.39; –0.23]
Mild side effects, 1 in 10	0.00	0.00	0.04
	[–0.08; 0.08]	[–0.09; 0.08]	[–0.03; 0.11]
Severe side effects, 1 in 10k	0.24	0.27	0.24
	[0.15; 0.33]	[0.16; 0.38]	[0.17; 0.31]
Origin, Germany	–0.35	–0.63	–0.40
	[–0.50; –0.20]	[–0.79; –0.48]	[–0.51; –0.29]
Origin, U.K.	–0.37	–0.58	–0.37
	[–0.53; –0.23]	[–0.75; –0.42]	[–0.47; –0.27]
Origin, U.S.	–0.29	–0.58	–0.44
	[–0.43; –0.15]	[–0.74; –0.43]	[–0.54; –0.35]
Endorsed by Gates Foundation	–0.16	–0.11	–0.16
	[–0.23; –0.08]	[–0.16; –0.05]	[–0.21; –0.11]
Endorsed by Oxford	–0.14	–0.11	–0.14
	[–0.20; –0.07]	[–0.18; –0.05]	[–0.19; –0.09]
Endorsed by CDC	–0.18	–0.12	–0.29
	[–0.27; –0.10]	[–0.17; –0.06]	[–0.36; –0.23]
Endorsed by WHO	–0.26	–0.05	–0.10
	[–0.34; –0.19]	[–0.12; 0.00]	[–0.15; –0.04]
Participants	832	1,474	1,001
Observations	8,320	14,740	10,010

Notes: Each estimate shows results for a combination of survey country and model. The first number gives the mean estimate, the range below the 95% confidence interval. This table omits individual-level covariates. The full table is S2 Table.

Recovering the findings in Kreps *et al*.’s study [10], we find that changes in the probability of minor side effects do not significantly impact decisions, whereas vaccine efficacy and the risk of severe side effects do. In terms of country of origin, the results align closely across the three survey countries, with Germany, the U.K., and the U.S. being perceived similarly in contrast to China (excluded category), but with minimal differences among themselves. Interestingly, unlike in Kreps et al’s study, we do not observe a consistently strong preference for vaccines with longer durations of effectiveness [10]. This discrepancy might be attributed to the higher COVID-19 case numbers during our survey period compared to when Kreps *et al*. conducted their experiment (July 2020) [10]. Overall, these findings suggest that respondents were attentive to the vaccine attributes in our design and responded in ways that are consistent with previous research. Illustrative substantive effect size simulations can be seen in S1 Fig, obtained in a manner similar to how we calculate them for endorsers described below.

### Simple endorsements

We first consider the effect of a WHO endorsement on people’s willingness and speed to take the vaccine and compare it to endorsements from other actors active in global health governance. Table 2 from above presents the coefficient estimates for each of the four potential endorsers. Compared to an absence of endorsement, a WHO endorsement statistically significantly reduces the time until one takes the vaccine across all three survey countries, even after accounting for the realizations of other endorsers. In Canada, the magnitude of this effect is comparable to increasing vaccine efficacy from 50% to 90% although the effects are somewhat smaller in Japan and the United States. Similar patterns are observed for endorsements from the CDC, Oxford, and the Gates Foundation across the three survey countries.

These results are significant for two reasons. First, they demonstrate that each endorser’s support can accelerate vaccination intentions, contributing to the quicker resolution of a health crisis. This is a notable extension of previous research, which focused on general willingness to vaccinate without differentiating timing [10,12,13]. Second, the findings suggest that each endorser’s influence remains potent even in the presence of multiple (non-)endorsements, suggesting that prior endorsement results are robust to alternative designs.

**Fig 1:**
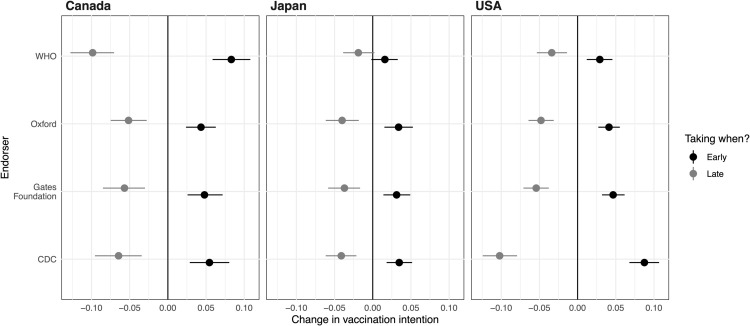
Substantive effects of endorsements on willingness to take vaccine. This figure shows the effects when a given entity endorses a vaccine compared to when it does not do so, showing the substantive effect of endorsement relative to when an individual indicates they will get vaccinated. The figure is constructed like S1 Fig.

To effectively compare the impact of WHO endorsements with other entities’, we need to examine the magnitudes of their effects. However, interpreting effect sizes based on coefficients in ordered probit models is not straightforward. Therefore, we use simulations of substantive effects [48]. Specifically, we calculate the differences in probabilities for each outcome choice that result from altering one attribute at a time, while averaging over all other vaccine attributes and demographic factors. Fig 1 illustrates these probability changes for both the “late” and “early” vaccination categories across each country (represented in the columns of panels), based on the three models in Table 2. We omit the middle category from this visual presentation for clarity and simplicity as it is just the remainder.

In Fig 1, the x-axis displays changes in the probability of a respondent choosing “late” (gray dot/ line) or “early” (black) when there is an endorsement from each entity on the y-axis, averaging over all covariates. The results show that the WHO endorsements reduce the “late" probability and increases the “early" probability by similar amounts. These effects are most pronounced for Canada, followed by the United States. In Canada, the effect of WHO endorsements is the greatest compared to the other three, whereas it ranks among the smallest in Japan and the United States. Overall, these results provide no consistent evidence that the WHO is the most critical or influential endorser at least in terms of encouraging vaccine uptake. Other entities, such as Oxford University, the Gates Foundation, and the CDC demonstrate similar or sometimes greater influence. All can contribute to increases in vaccination intentions and speed.

### WHO endorsement and other endorsements

Next, we study whether WHO endorsements are more or less consequential under varying numbers of endorsements from other entities. To examine this, we first replace the endorsement indicators for the CDC, Oxford, and the Gates Foundation with a cumulative count of their endorsements. This sum is then included alongside an interaction term with the WHO endorsement. Table 3 presents the coefficient estimates for the WHO endorsement, the number of endorsements from other entities, their interaction, and other vaccine features; the full table of coefficient estimates is available in the S4 Table.

**Table 3:** Estimates for vaccine uptake models interacting WHO endorsement and number of other endorsers.

	Canada	Japan	USA
Protection duration, 5 years	–0.04	–0.08	–0.07
	[–0.11; 0.03]	[–0.15; –0.01]	[–0.12; –0.02]
Efficacy, 50%	0.17	0.22	0.23
	[0.06; 0.29]	[0.15; 0.30]	[0.16; 0.30]
Efficacy, 90%	–0.29	–0.07	–0.32
	[–0.39; –0.19]	[–0.15; 0.00]	[–0.39; –0.24]
Mild side effects, 1 in 10	0.00	0.00	0.04
	[–0.08; 0.08]	[–0.08; 0.08]	[–0.02; 0.11]
Severe side effects, 1 in 10k	0.24	0.27	0.24
	[0.15; 0.34]	[0.16; 0.39]	[0.17; 0.30]
Origin, Germany	–0.36	–0.63	–0.39
	[–0.52; –0.21]	[–0.77; –0.49]	[–0.50; –0.29]
Origin, U.K.	–0.39	–0.58	–0.37
	[–0.53; –0.24]	[–0.74; –0.43]	[–0.47; –0.27]
Origin, U.S.	–0.29	–0.58	–0.44
	[–0.42; –0.15]	[–0.73; –0.44]	[–0.53; –0.34]
Endorsed by WHO	–0.59	–0.15	–0.19
	[–0.78; –0.40]	[–0.34; 0.04]	[–0.32; –0.05]
Endorsed by WHO	0.20	0.06	0.06
× Other endorsements	[0.09; 0.32]	[–0.06; 0.18]	[–0.02; 0.14]
Other endorsements	–0.27	–0.14	–0.23
	[–0.34; –0.19]	[–0.22; –0.07]	[–0.28; –0.17]
Participants	832	1,474	1,001
Observations	8,320	14,740	10,010

Notes: Each estimate shows results for a combination of survey country and model. The first number gives the mean estimate, the range below the 95% confidence interval. This table omits individual-level covariates. The full table is S4 Table.

Across the three survey countries, we find a diminishing effect of WHO endorsements as the number of endorsements from other entities increases, as indicated by the positive coefficient on the interaction term. This suggests that WHO endorsements are substitutable—being more influential when there are fewer endorsements from other entities. Conversely, the presence of other endorsers can diminish the WHO’s importance, a case in which the WHO endorsement is less needed (as indicated by our previous analyses), however. This pattern is more distinct in Canada and the United States, while in Japan, the estimates are less clear-cut.

**Fig 2:**
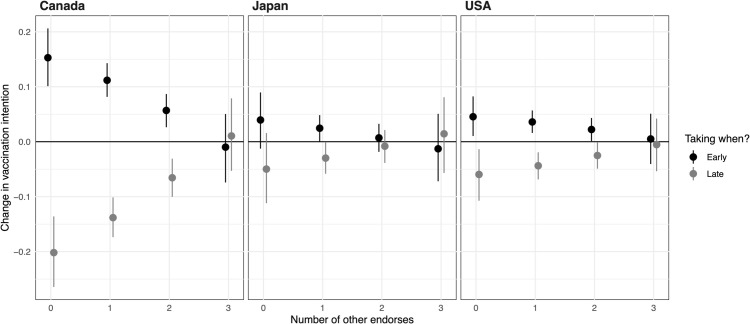
WHO effects conditional of the number of other endorsements. This figure shows how multiple endorsers affect when people will get vaccinated. Given WHO endorsement, the figure shows change in probability for taking the vaccine early (gray) or late (black) based on the number of co-endorsers.

Fig 2 shows the substantive effects of the WHO endorsement on each of the outcome probability (y-axis) conditional on the number of other endorsements (x-axis). In Canada and the United States, we clearly observe the effect moderation by the number of other endorsements. When no other entities endorse a vaccine, the WHO’s endorsement increases the probability of getting vaccinated “early” and decreases the probability of “late” vaccination. However, as more endorsements are added along the x-axis, the influence of the WHO on vaccination intentions diminishes. Notably, the substantial effect of the WHO endorsement actually becomes indistinguishable from zero when the CDC, Oxford University, and the Gates Foundation jointly endorse a vaccine. In Japan, people react rather little to the WHO endorsement conditional on any number of other endorsers. This suggests that the absence of an effect for Japan in Fig 1 was not due to effect heterogeneity with regard to the number of other endorsers.

Our findings, particularly in Canada and the United States, suggest that the WHO’s endorsement can make the largest difference when other entities are slower to examine and endorse a vaccine. Conversely, if the WHO is slow to endorse, other entities can effectively step in to fill the gap. More generally, this substitutability implies that a multitude of endorsements or a consensus among various health-focused organizations is not necessarily crucial for vaccine promotion. Instead, the key is to ensure that the first or first few credible entities take prompt action, and the WHO can be key if it moves fast and early.

To illustrate this point, we simulate the expected share of vaccinated people—quantities that are more relevant from a public health perspective—as time progresses under different endorsement scenarios: with and without the WHO’s endorsement and with zero or three other endorsements. We reestimate the models from Table 3 but rely on the full range of outcome options so that we can show the vaccinated shares over time. For every observation in the data, we simulate when they would take each type of vaccine, which lets us calculate the share of vaccinated people (measured by stated vaccination intention) at each point in time. Relying on the parametric bootstrap, we obtain measure of uncertainty based on the model.

**Fig 3:**
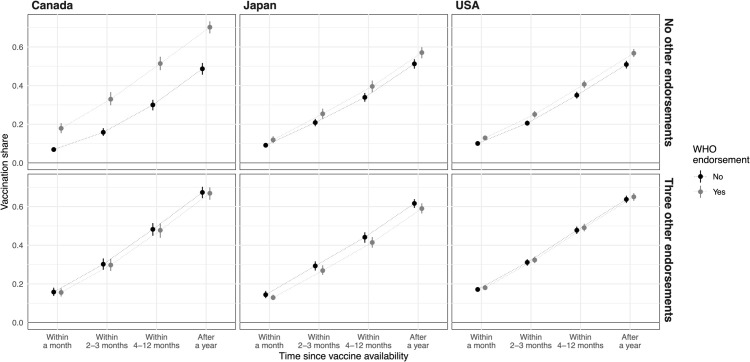
WHO effects on the share of vaccinated people when there are no or two other vaccine endorsements. In each row of panels, the x-axis gives the time points and the y-axis shows the share of vaccinated people when there is WHO endorsement (gray) and when there is not (black). The upper panels give the results assuming no other vaccine endorsements, the bottom assuming three others. The dot gives the median estimate, the line 68% confidence interval (about one standard deviation).

In Fig 3, the y-axis represents the share of vaccinated individuals at a particular time point (x-axis). For example, in Canada, when there are no other endorsements (upper panel), about 18% of the population will have taken the WHO-endorsed vaccine (gray) within a month, 32% within 2-3 months, 51% within 4-12 months, and 69% after a year. When the WHO does not endorse the vaccine (black), these shares drop significantly, aligning with our earlier discussion.

However, when there are endorsements from three other entities (lower panel), the vaccination rates reach about the same as when there is only a WHO endorsement (gray in upper panel). This occurs even without the WHO endorsement because the other two endorsements have already increased vaccination rates while reducing the impact of an additional WHO endorsement. This demonstrates the significant role of timely endorsements from credible entities in promoting vaccination and the diminishing impact of additional endorsements once initial credibility is established.

### WHO endorsement for low-quality vaccines

Conjoint analyses of vaccination intentions are powerful because they allow us to study a large space of possible vaccines. Of course, the vast space of potential vaccines does not exist in reality among the vaccines that make it into medical trials. In November 2020, the world learned the remarkable effectiveness of mRNA vaccines against COVID-19 with minuscule side effects. When vaccine trials started earlier that year, the U.S. Federal and Drug Administration said it would be willing to approve a vaccine with 50% effectiveness against severe illness, a benchmark that was eclipsed by the Phase 3 trial results by BioNTech/Pfizer and Moderna vaccines in November 2020 [49].

Vaccines developed for future pandemics may not always be highly effective, particularly those rapidly produced in the early stages of an outbreak due to urgent public health demands. Existing research examining vaccination intentions using vaccine attribute profiles consistently finds that respondents prefer higher-quality vaccines, reducing the relative importance of endorsements in such cases. Consequently, it is crucial to examine the influence of WHO endorsements specifically for lower-quality vaccines, as vaccination intentions for these vaccines are likely to be more sensitive to credible endorsements.

**Fig 4:**
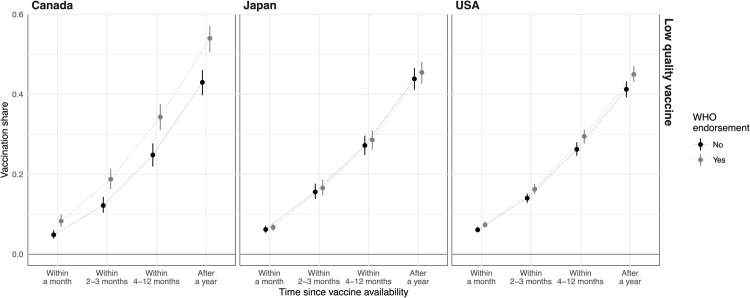
WHO effects on the share of vaccinated people for low-quality vaccine. The x-axis gives the time points and the y-axis shows the share of vaccinated people when there is WHO endorsement (gray) and when there is not (black). The dot gives the median estimate, the line 68% confidence interval (about one standard deviation).

We use simulations based on the models without interactions and with the full range of outcomes to illustrate the influence of WHO endorsements on the share of vaccinated people over time, as shown in Fig 4. We define low-quality vaccines as those with shorter protection duration (1 year), lowest efficacy (50%), and higher odds of side effects (1 in 10,000 and 1 in 30 for severe and mild side effects, respectively). We average over the randomized country of origin and endorsements by the CDC, Oxford, and Gates foundation.

Fig 4 presents simulation results showing vaccination shares over time, comparing scenarios with a WHO endorsement (gray) to those without (black) like before. In Japan, the WHO endorsement has little effect on vaccination rates at each time point for low-quality vaccines, consistent with previous observations. In the United States, there is a small effect on vaccination rates that gradually increases over time, though the increase is noisy. The Canadian case is particularly instructive. When people are receptive to WHO endorsements, we observe a distinctly higher vaccination rate over time. After a year, there is a ten percentage-point increase in the final share of individuals opting for the WHO-endorsed vaccine.

## Limitations

Our work is not without methodological limitations. We recognize as a key limitation of our study that it was conducted at a specific moment—late 2020, immediately prior to the public availability of COVID-19 vaccines—thus capturing a unique period of heightened uncertainty about vaccination. As a result, the external validity of our findings is necessarily limited: endorsement effects observed under conditions of novelty and uncertainty may not fully generalize to later stages of the pandemic, when vaccines had become more widely available and politicized, or to more routine vaccination contexts (e.g., seasonal influenza, RSV). At the same time, this temporal context is conceptually important. In late 2020, citizens were forming expectations about unprecedented vaccine technologies, governments were preparing mass immunization campaigns, and uncertainty about safety and efficacy was at its peak. Expert endorsements from trusted institutions were therefore likely to play an outsized role in shaping public attitudes at precisely the point when those attitudes were most malleable and consequential for subsequent uptake. We encourage future research to extend this framework by systematically testing how endorsement effects evolve across different phases of a pandemic and in non-pandemic vaccination settings.

Relatedly, our study is necessarily limited in the range of actors examined as potential vaccine endorsers. We deliberately restricted attention to organizations that were widely recognized across Canada, Japan, and the United States in late 2020 and that represent distinct institutional bases of legitimacy (international organization, national public health authority, academic institution, philanthropic foundation). This design choice ensured cross-national comparability and allowed us to probe how endorsements grounded in different forms of legitimacy might complement or substitute for one another. At the same time, this focus does not capture the full diversity of actors that shape vaccine discourse. Country-specific organizations such as national medical associations, local NGOs (e.g., the March of Dimes in the United States), and grassroots pro- or anti-vaccination groups may exert important influences on vaccine attitudes within particular contexts. Likewise, commercial actors such as vaccine manufacturers or other business entities play a prominent role in shaping perceptions of safety and trustworthiness. Although we do not have strong theoretical reasons to expect systematically different dynamics from including these actors, our findings should nonetheless be understood within these scope conditions in mind.

Nonetheless, the methodological contribution of our design lies precisely in clarifying these boundaries and demonstrating how conjoint experiments can systematically evaluate the persuasive power of diverse endorsers. Future research should build on this design by expanding the range of endorsing actors, contrasting local, domestic, regional, and global organizations, and incorporating commercial as well as oppositional voices. Examining whether endorsements from local professional associations, grassroots campaigns, or corporate actors operate in supportive, competitive, or counterproductive ways relative to international organizations like the WHO would provide a more comprehensive picture.

Additionally, we note that the data for each of our cross-national surveys were derived from online opt-in platforms. As we discussed earlier, such panels are widely used in experimental studies of vaccine uptake but inevitably involve a tradeoff between response quality and demographic representativeness. Recent work by Stagnaro *et al*. finds that while platforms like Prolific (which provided our Canadian and US samples) is among the least demographically representative services, it nevertheless provide very high-quality data, successfully replicating well-studied experimental effects and demonstrating high levels of attentiveness [50]. Our work makes an effort to strike a balance between quality and representativeness by calculating and applying post-stratification weights to our data. This procedure improved the alignment of our samples with national benchmarks, though a few residual imbalances remain. In light of validation evidence from recent research [43–45], we are cautiously confident that our main findings are not artifacts of sample composition. Nonetheless, we hope that future work will make an effort to replicate the effects studied in this piece in a diverse range of both probability and non-probability samples.

Finally, consistent with the scope and theoretical focus of this paper, we deliberately refrain from conducting subgroup analyses of whether individual characteristics moderate treatment effects, or from probing underlying mechanisms directly. Such analyses would require distinct research designs guided by specific theoretical expectations. Our aim here is to take a first crucial step in establishing the effects of IO endorsements within a complex and diversified global health landscape. We hope that these findings will provide a foundation for future work to extend this framework, both by investigating the mechanisms through which endorsements shape attitudes and by examining theoretically motivated moderating roles of individual-level attributes such as age, ideology, and education.

## Conclusion

Our conjoint experimental analysis provides novel insights into the critical role that global expert entities can play in promoting vaccine acceptance within an increasingly diversified, multi-actor global health governance landscape. We find that WHO endorsements, alongside the three other public health organizations examined in this study, are associated with a statistically significant, cross-national reduction in vaccine hesitancy, measured as the delay between vaccine availability and willingness to receive it. Our timing-based measure is a meaningful, yet under-studied, dimension of vaccine uptake that directly speaks to the urgency of public health communication during a pandemic.

Our findings suggest that the WHO does not hold a uniquely privileged position in shaping public opinion on global health issues. Endorsements from other prominent actors—national health agencies, academic institutions, and philanthropic foundations—can meaningfully influence vaccine acceptance and, in some cases, “crowd out" the marginal effect of a WHO endorsement. When credible signals from these actors are already available, an additional WHO endorsement exerts only modest influence, implying that multiple respected voices may sufficiently shape public opinion on their own. At the same time, this pattern reflects the broader political context in which IOs like the WHO operate. As organizations shaped by member states’ agendas and institutional interests, IOs often communicate in politicized environments that can complicate their ability to claim unique authority [31]. While prior evidence on whether politicization diminishes IO influence is mixed [29,32], our results indicate that, in the specific domain of vaccine promotion, the influence commonly attributed to the WHO can be effectively supplemented—or even substituted—by other credible global health actors.

These conditional patterns carry important implications for global health communication strategies. First, they highlight the value of pluralization: mobilizing multiple authoritative actors—international, national, academic, and philanthropic—can collectively shape vaccine acceptance, reducing reliance on any single source. Second, they underscore the importance of synchronization: timely endorsements are critical, since a delayed WHO endorsement adds little once other respected actors have already spoken. Conversely, WHO communication is likely to be most consequential in moments of uncertainty, when alternative voices are absent or delayed, or when vaccines are perceived as less effective or safe. In such contexts, WHO endorsement can fill informational vacuums by helping to reduce hesitancy and accelerate uptake. More broadly, our results suggest that effective communication in global health crises is less about singular authority than about coordination, timing, and complementarity among credible voices.

Additionally, our study makes a methodological contribution by utilizing a novel measure of vaccine hesitancy [24]. Rather than employing a binary yes/no measure of willingness to vaccinate, we use an ordinal scale capturing the expected delay between vaccine availability and uptake. This timing-based approach is an important yet under-studied indicator of vaccine hesitancy, offering nuanced insight into both the likelihood and timing of vaccine acceptance. Given the importance of rapid vaccine uptake during pandemics, this measure provides valuable practical insights.

Our experiment provides evidence that an IO can influence public attitudes on issues with significant personal implications, an area relatively understudied in existing IO research, with notable exceptions [51,52]. Prior studies primarily focus on IO effects regarding government policy attitudes, particularly in foreign and security domains [53–56]. In contrast, our findings demonstrate that WHO endorsements influence individuals’ decisions about personal health behaviors, specifically vaccine uptake timing [30,57,58]. However, we acknowledge these effects must be interpreted within the broader multi-actor landscape of global health governance. Thus, our work contributes to understanding the impact of IOs in comparison to or in combination with other global governance entities.

## Supporting information

S1 File Instrument flow. A detailed overview of the survey for a participant.
(PDF)

S1 Fig Sample balance. Each panel presents covariate-specific standardized differences in means/proportions between the sample and the target population, reported both prior to and following the application of survey weights for each country.
(TIFF)

S1 Appendix Manipulation Checks: China Treatment for WHO. A detailed discussion of the manipulation checks of the China treatment for the WHO.
(PDF)

S1 Table Estimates for manipulation check models. (PDF)

S2 Table Estimates for vaccine uptake models. (PDF)

S3 Table Estimates for vaccine uptake models that include indicators for China manipulation. (PDF)

S4 Table Estimates for vaccine uptake models interacting WHO endorsement and number of other endorsers. (PDF)

S2 Fig Substantive effects for vaccine attributes. This figure gives the effects of changes to the efficacy and safety profile as well as to the country of origin. The black dots and lines give the changes to the probability to taking the vaccine late, and the gray counterparts to the probability of taking it swiftly. Lines are 95% confidence intervals.
(PDF)
