# Supplementary material for: Assessing WHO’s influence: A randomized conjoint experiment on vaccine endorsements in diversified global health systems
Source: PLOS Glob Public Health. 2025 Nov 21;5(11):e0005410. doi: 10.1371/journal.pgph.0005410 (PMC12637889; doi:10.1371/journal.pgph.0005410)
Supplement: S1 File — A detailed overview of the survey for a participant. (PDF) [file pgph.0005410.s001.pdf]

S1 File. Instrument flow

The following outlines the flow through the survey for a participant.

1. Introduction, consent

2. Pre-treatment covariates, Part 1

   (a) "We'd like to get your feelings toward a number of countries and organizations on a 'feeling thermometer.' A rating of zero degrees means you feel as cold and negative as possible. A rating of 100 degrees means you feel as warm and positive as possible. You would rate the group at 50 degrees if you don't feel particularly positive or negative toward the entity." Countries and organizations are: "Argentina"; "China"; "Denmark"; "North Atlantic Treaty Organization (NATO)"; "United Nations Children's Fund (UNICEF)"; "World Health Organization (WHO)". Slider going from 0 (Cold) to 100 (Warm).

   (b) "How frequently do you take flu vaccination?" *Every year; Most years; Some years; Never*.

   (c) "All in all, are you currently satisfied or unsatisfied with the job that the national government is doing?" *Very satisfied; Somewhat satisfied; Neither satisfied nor unsatisfied; Somewhat unsatisfied; Very unsatisfied*.

   (d) "Every country in the world is currently dealing with the novel coronavirus. Taken all together, how would you rate the situation regarding Covid-19 in your country" *Very bad; Somewhat bad; Neither bad nor good; Somewhat good; Very good*.

   (e) "Think of a ladder with 10 steps representing where people stand in your country. At step 10 are people who are best off – those who have the most money, the most education, and the most respected jobs. At step 1 are the people who are worst off – those who have the least money, least education, and the least respected jobs. Where would you place yourself on this ladder?" *Step 1 (lowest); Step 2; ...; Step 9; Step 10 (highest)*.

   (f) "Please consider the following statements and tell us to which extent you agree or disagree with it." Statements are: "Adults should get all recommended vaccines."; "Children should get all recommended vaccines."; "Generally speaking, recommended vaccines are safe." For each: *1 (Strongly agree); 2; ...; 6; 7 (Strongly disagree)*.

3. Distraction task, asking about "last major meal you had."

4. Pre-treatment covariates, Part 2. The wording are country-specific. Below is the U.S. version as an example.

   (a) "In what year were you born?" Integer entry.

   (b) "Are you ..." *Man; Woman; Other*.

(c) "What is the highest level of education you have completed?" *No high school; High school graduate; Some college; 2-year college; 4-year college; Post-graduate*.

(d) "In general, how would you describe your own political viewpoint?" *Very liberal; Liberal; Moderate; Conservative; Very conservative; Not sure*.

5. Introducing potential endorsers; China/WHO treatment. On top of the page, we provide the following introduction:

> As you may know, scientists around the world are working to develop a vaccine for COVID-19. On the next five pages, we will show you several hypothetical vaccines that differ in several ways. We will ask you to assess how long you would wait to take each, if you would take a vaccine at all. One key aspect in which these hypothetical vaccines will differ is which entities endorse the vaccine. Below, you see all entities that could endorse the vaccine. Please read the background for each carefully and tell us how you feel toward each."

   Below each entity's introductory phrases, we ask, "How do you feel about ...?". Answer options are a slider from 0 (Cold) to 100 (Warm), as before.

   - "The Bill & Melinda Gates Foundation is the world's largest private foundation which has been working on infectious diseases for decades."
   - "Oxford University is the third oldest leading university in the world with researchers that have been developing several vaccines for various infectious diseases for many decades."
   - "The World Health Organization (WHO) is the world's most important international organization dedicated to all matters of health." If the China treatment is applied, the following is appended: "In the early days of the current Coronavirus pandemic, the WHO was sharply criticized for its deference to China where the novel coronavirus originated."
   - "The Center for Disease Control (CDC) is the world's premier public health and infectious disease bureaucracy. It is located in the United States of America.

6. Five screens of conjoint experiments. The vaccine profile pairs are introduced by stating "You find two hypothetical vaccines described in the table below. Please examine them carefully and then answer the questions below. For each of the two vaccines, we ask, "If Vaccine A were to become available at no cost to you, would you take Vaccine A?" *No, never; Yes, within a month"; "Yes, within 2-3 months"; "Yes, within 4-12 months; "Yes, after a year"*.

7. Debriefing and thank-you page.
