# Supplementary material for: Assessing WHO’s influence: A randomized conjoint experiment on vaccine endorsements in diversified global health systems
Source: PLOS Glob Public Health. 2025 Nov 21;5(11):e0005410. doi: 10.1371/journal.pgph.0005410 (PMC12637889; doi:10.1371/journal.pgph.0005410)
Supplement: S1 Appendix — A detailed discussion of the manipulation checks of the China treatment for the WHO. (PDF) [file pgph.0005410.s003.pdf]

S1 Appendix. Manipulation Checks: China Treatment for WHO

We seek to examine whether our China-related treatments—the tainting of the WHO—worked. Specifically, we test whether people's feelings toward the WHO changes significantly when exposed to the cue about a mention of China in the WHO's introduction.

In the sequence of the experiment, we treat people (or not) with the information that China's actions may have tainted the WHO. Specifically, recall that we introduce the Gates Foundation, CDC, Oxford University, and the WHO via short texts, which may include the China treatment for the WHO. Immediately below the respective texts, we asked about the warm/ cold feelings a survey-taker has about the organizations. Responses were recorded via a slider, going from 0 (Cold) to 100 (Warm). Since this we recorded immediately below the treatment, we see this an easy manipulation check.

For each survey-taker and separate by country, we regress the feeling score for the WHO on the China-treatment indicator, all demographic covariates spelled out above, and the feeling thermometer scores for China and WHO collected at the very beginning of the experiment. We use a linear regression as well as an ordered probit to allow for a more flexible, non-linear handling of the feelings toward the WHO. We used 20, 40, 60, and 80 as breaks for the ordinal variable.

S1 Table shows the coefficient estimates of the China treatment in the feeling thermometer linear (columns 2, 4, 6) and ordinal probit (columns 3, 5, and 7) models. The linear model for Japan shows a tiny effect, namely a change of -2.84 on a 0-100 feeling thermometer scale. The coefficient is statistically insignificant in the ordered probit model. In the U.S. samples, the coefficients are statistically insignificant, and even the point estimate in the linear model is miniscule. In contrast, the statistical significance for the Canadian sample is clear and consistent across both estimators. Learning about the WHO catering to China lowers Canadian's feeling toward the WHO. However, the magnitude of the effect in the linear model is also tiny.

The BIC as a model selection criterion prefers for the U.S. and Japan a model in which the WHO endorsement does not depend on the China treatment over one that accounts for it; in the Canada data, it is the other way around. See S2 Table and S3 Table.

Thus, we judge our China-treatment to be weak in the statistically strongest and consistent case (Canada) and even weaker and statistically inconsistent in the other cases (U.S., Japan). Recall that the feeling thermometer scores were measured immediately after the treatment and that having the pre-treatment variant of the outcome to increase precision. Yet, we find at best faint changes in the feelings toward the WHO.

We can speculate that the public was already "pre-treated" about the WHO, having already internalized the real-world negative messaging about the WHO. In that case, our China treatment would not provide new information. However, politicization of the WHO differed between Canada, the United States, and Japan, casting doubt on this explanation. Future research could consider either a stronger treatment, which would raise questions about whether one would encounter such a strong treatment in the real-word. For our purposes, we will forego making comparisons for the conjoint effects that distinguish between the China treatments, thereby not testing Hypothesis 2. S3 Table gives the conjoint results we discuss below while considering the China treatment.
