# Supplementary material for: Assessing WHO’s influence: A randomized conjoint experiment on vaccine endorsements in diversified global health systems
Source: PLOS Glob Public Health. 2025 Nov 21;5(11):e0005410. doi: 10.1371/journal.pgph.0005410 (PMC12637889; doi:10.1371/journal.pgph.0005410)
Supplement: S1 Table — (PDF) [file pgph.0005410.s004.pdf]

S1 Table. Estimates for manipulation check models.

	Canada		Japan		United States	
WHO/China	-5.17	-0.80	-2.84	-0.02	-0.60	0.19
	[-7.11; -3.19]	[-1.18; -0.42]	[-4.97; -0.74]	[-0.19; 0.16]	[-2.31; 1.15]	[-0.12; 0.51]
FT, WHO (earlier)	0.84	0.15	0.41	0.03	0.87	0.16
	[0.80; 0.88]	[0.13; 0.17]	[0.35; 0.48]	[0.03; 0.04]	[0.83; 0.90]	[0.14; 0.19]
FT, China	-0.04	0.00	0.24	0.02	0.00	0.00
	[-0.08; 0.01]	[-0.01; 0.01]	[0.18; 0.30]	[0.01; 0.02]	[-0.04; 0.03]	[-0.01; 0.01]
Vaccine attitude	-1.01	-0.26	-1.53	0.01	-1.17	0.01
	[-2.26; 0.15]	[-0.48; -0.02]	[-2.87; -0.25]	[-0.16; 0.19]	[-2.19; -0.25]	[-0.17; 0.18]
Gender, male	-0.36	0.28	-4.62	-0.49	-0.45	0.26
	[-2.35; 1.75]	[-0.07; 0.65]	[-7.02; -2.35]	[-0.71; -0.29]	[-2.24; 1.35]	[-0.05; 0.58]
Education, university	-3.02	0.26	-5.92	-0.47	-2.34	-0.17
	[-5.05; -1.04]	[-0.14; 0.64]	[-8.47; -3.28]	[-0.69; -0.24]	[-4.16; -0.51]	[-0.50; 0.16]
Age	0.07	0.01			-0.04	0.00
	[-0.01; 0.16]	[0.00; 0.03]			[-0.11; 0.02]	[-0.01; 0.01]
Age, less than 30			2.97	0.03		
			[-0.98; 6.77]	[-0.32; 0.38]		
Age, more than 60			3.13	0.35		
			[0.72; 5.63]	[0.12; 0.57]		
Age, no answer			-6.06	-0.06		
			[-16.76; 4.90]	[-0.77; 0.66]		
Ideology, DKNO			-8.85	-0.46	-3.77	0.62
			[-15.80; -2.06]	[-1.04; 0.10]	[-9.42; 1.93]	[-0.27; 1.51]
Ideology	-5.75	-0.67	-15.89	-1.35	-4.89	-0.60
	[-10.66; -0.84]	[-1.48; 0.09]	[-23.48; -8.74]	[-2.26; -0.50]	[-8.64; -1.18]	[-1.30; 0.09]
Intercept	14.84		33.71		14.24	
	[8.77; 20.98]		[27.87; 39.48]		[9.54; 18.84]	
Cut point 1		3.25		-0.56		3.62
		[2.10; 4.37]		[-1.20; 0.06]		[2.61; 4.65]
Cut point 2		5.88		0.75		6.10
		[4.52; 7.24]		[0.13; 1.38]		[4.89; 7.36]
Cut point 3		9.03		2.54		9.62
		[7.39; 10.64]		[1.88; 3.21]		[8.06; 11.20]
Cut point 4		12.90		6.27		13.26
		[10.87; 14.88]		[5.09; 7.47]		[11.36; 15.31]
Observations	832	832	1,474	1,474	1,001	1,001
Model	Linear	O Probit	Linear	O Probit	Linear	O Probit
