# Supplementary material for: Assessing WHO’s influence: A randomized conjoint experiment on vaccine endorsements in diversified global health systems
Source: PLOS Glob Public Health. 2025 Nov 21;5(11):e0005410. doi: 10.1371/journal.pgph.0005410 (PMC12637889; doi:10.1371/journal.pgph.0005410)
Supplement: S2 Table — (PDF) [file pgph.0005410.s005.pdf]

S2 Table. Estimates for vaccine uptake models.

	Canada	Japan	USA
Protection duration, 5 years	-0.05	-0.08	-0.07
	[-0.12; 0.02]	[-0.15; -0.01]	[-0.12; -0.02]
Efficacy, 50%	0.17	0.22	0.23
	[0.04; 0.29]	[0.14; 0.29]	[0.16; 0.30]
Efficacy, 90%	-0.29	-0.07	-0.32
	[-0.39; -0.19]	[-0.15; 0.01]	[-0.40; -0.24]
Mild side effects, 1 in 10	-0.01	0.00	0.04
	[-0.09; 0.08]	[-0.09; 0.08]	[-0.02; 0.11]
Severe side effects, 1 in 10k	0.24	0.27	0.24
	[0.15; 0.34]	[0.16; 0.38]	[0.17; 0.31]
Origin, Germany	-0.37	-0.63	-0.40
	[-0.52; -0.21]	[-0.77; -0.48]	[-0.51; -0.29]
Origin, U.K.	-0.38	-0.58	-0.37
	[-0.53; -0.23]	[-0.74; -0.43]	[-0.47; -0.27]
Origin, U.S.	-0.29	-0.58	-0.44
	[-0.44; -0.15]	[-0.73; -0.43]	[-0.54; -0.33]
Endorsed by Gates Foundation	-0.16	-0.11	-0.16
	[-0.23; -0.08]	[-0.16; -0.05]	[-0.21; -0.11]
Endorsed by Oxford	-0.14	-0.12	-0.14
	[-0.21; -0.08]	[-0.18; -0.05]	[-0.19; -0.09]
Endorsed by CDC	-0.18	-0.12	-0.29
	[-0.27; -0.09]	[-0.18; -0.06]	[-0.36; -0.23]
Endorsed by WHO	-0.27	-0.05	-0.10
	[-0.35; -0.19]	[-0.11; 0.01]	[-0.16; -0.04]
Vaccine attitude	0.10	0.22	0.13
	[-0.06; 0.26]	[0.10; 0.34]	[0.05; 0.21]
Gender, male	-0.36	-0.21	-0.44
	[-0.64; -0.08]	[-0.43; 0.01]	[-0.58; -0.29]
Education, university	-0.02	0.08	-0.28
	[-0.28; 0.24]	[-0.18; 0.34]	[-0.43; -0.13]
Age	0.00		0.01
	[-0.01; 0.01]		[0.00; 0.01]
Age, less than 30		-0.20	
		[-0.44; 0.03]	
Age, more than 60		-0.06	
		[-0.30; 0.17]	
Age, no answer		3.05	
		[2.70; 3.40]	
Ideology, DKNO		-0.05	0.05
		[-0.50; 0.38]	[-0.51; 0.61]
Ideology	0.08	-0.48	0.81
	[-0.44; 0.60]	[-1.11; 0.16]	[0.55; 1.09]
Cut point 1	-1.15	-1.17	-0.72
	[-1.70; -0.61]	[-1.61; -0.74]	[-0.99; -0.46]
Cut point 2	-0.65	-0.74	-0.23
	[-1.21; -0.11]	[-1.16; -0.31]	[-0.50; 0.03]
Participants	832	1,474	1,001
Observations	8,320	14,740	10,010
