# Supplementary material for: Assessing WHO’s influence: A randomized conjoint experiment on vaccine endorsements in diversified global health systems
Source: PLOS Glob Public Health. 2025 Nov 21;5(11):e0005410. doi: 10.1371/journal.pgph.0005410 (PMC12637889; doi:10.1371/journal.pgph.0005410)
Supplement: S3 Table — (PDF) [file pgph.0005410.s006.pdf]

S3 Table. Estimates for vaccine uptake models that include indicators for China manipulation.

	Canada	Japan	USA
Protection duration, 5 years	-0.04	-0.08	-0.07
	[-0.12; 0.02]	[-0.15; -0.02]	[-0.12; -0.02]
Efficacy, 50%	0.17	0.22	0.23
	[0.05; 0.29]	[0.15; 0.29]	[0.15; 0.30]
Efficacy, 90%	-0.30	-0.07	-0.32
	[-0.39; -0.19]	[-0.14; 0.01]	[-0.40; -0.24]
Mild side effects, 1 in 10	-0.01	0.00	0.04
	[-0.09; 0.08]	[-0.08; 0.09]	[-0.03; 0.10]
Severe side effects, 1 in 10k	0.24	0.27	0.24
	[0.14; 0.33]	[0.16; 0.38]	[0.17; 0.31]
Origin, Germany	-0.37	-0.63	-0.40
	[-0.51; -0.22]	[-0.78; -0.49]	[-0.50; -0.29]
Origin, U.K.	-0.38	-0.58	-0.37
	[-0.53; -0.24]	[-0.73; -0.42]	[-0.47; -0.27]
Origin, U.S.	-0.30	-0.58	-0.44
	[-0.43; -0.16]	[-0.74; -0.43]	[-0.54; -0.34]
Endorsed by Gates Foundation	-0.15	-0.11	-0.16
	[-0.23; -0.08]	[-0.16; -0.05]	[-0.21; -0.11]
Endorsed by Oxford	-0.15	-0.12	-0.14
	[-0.21; -0.08]	[-0.18; -0.05]	[-0.19; -0.09]
Endorsed by CDC	-0.18	-0.12	-0.30
	[-0.26; -0.09]	[-0.17; -0.06]	[-0.36; -0.23]
Not endorsed by WHO, China	0.14	-0.07	-0.07
	[-0.13; 0.41]	[-0.29; 0.17]	[-0.23; 0.09]
Endorsed by WHO, no China	-0.29	-0.10	-0.09
	[-0.39; -0.18]	[-0.19; -0.02]	[-0.16; -0.01]
Endorsed by WHO, China	-0.12	-0.07	-0.18
	[-0.39; 0.15]	[-0.30; 0.16]	[-0.34; -0.02]
Vaccine attitude	0.09	0.22	0.13
	[-0.07; 0.25]	[0.11; 0.34]	[0.05; 0.20]
Gender, male	-0.36	-0.22	-0.44
	[-0.62; -0.10]	[-0.43; 0.00]	[-0.58; -0.30]
Education, university	-0.02	0.09	-0.28
	[-0.28; 0.22]	[-0.18; 0.35]	[-0.43; -0.13]
Age	0.00		0.01
	[-0.01; 0.01]		[0.00; 0.01]
Age, less than 30		-0.21	
		[-0.45; 0.01]	
Age, more than 60		-0.05	
		[-0.29; 0.18]	
Age, no answer		3.05	
		[2.71; 3.41]	
Ideology, DKNO		-0.05	0.03
		[-0.48; 0.41]	[-0.53; 0.61]
Ideology	0.08	-0.48	0.81
	[-0.45; 0.57]	[-1.08; 0.14]	[0.56; 1.07]
Cut point 1	-1.07	-1.22	-0.76
	[-1.64; -0.52]	[-1.68; -0.77]	[-1.04; -0.48]
Cut point 2	-0.56	-0.78	-0.27
	[-1.11; -0.01]	[-1.23; -0.34]	[-0.54; 0.01]
Participants	832	1,474	1,001
Observations	8,320	14,740	10,010
