# Supplementary material for: Assessing WHO’s influence: A randomized conjoint experiment on vaccine endorsements in diversified global health systems
Source: PLOS Glob Public Health. 2025 Nov 21;5(11):e0005410. doi: 10.1371/journal.pgph.0005410 (PMC12637889; doi:10.1371/journal.pgph.0005410)
Supplement: S4 Table — (PDF) [file pgph.0005410.s007.pdf]

S4 Table. Estimates for vaccine uptake models interacting WHO endorsement and number of other endorsers.

	Canada	Japan	USA
Protection duration, 5 years	-0.04	-0.08	-0.07
	[-0.11; 0.03]	[-0.15; -0.01]	[-0.12; -0.02]
Efficacy, 50%	0.17	0.22	0.23
	[0.05; 0.29]	[0.15; 0.29]	[0.16; 0.31]
Efficacy, 90%	-0.29	-0.07	-0.31
	[-0.39; -0.20]	[-0.15; 0.01]	[-0.40; -0.24]
Mild side effects, 1 in 10	0.00	0.00	0.04
	[-0.08; 0.08]	[-0.09; 0.08]	[-0.02; 0.11]
Severe side effects, 1 in 10k	0.24	0.27	0.24
	[0.15; 0.34]	[0.16; 0.38]	[0.17; 0.31]
Origin, Germany	-0.36	-0.63	-0.39
	[-0.51; -0.22]	[-0.77; -0.48]	[-0.50; -0.29]
Origin, U.K.	-0.39	-0.58	-0.38
	[-0.54; -0.25]	[-0.74; -0.42]	[-0.48; -0.28]
Origin, U.S.	-0.29	-0.58	-0.44
	[-0.42; -0.15]	[-0.73; -0.43]	[-0.54; -0.34]
Endorsed by WHO	-0.59	-0.15	-0.18
	[-0.79; -0.40]	[-0.33; 0.05]	[-0.33; -0.04]
Endorsed by WHO $\times$ Other endorsements	0.21	0.06	0.06
	[0.10; 0.32]	[-0.06; 0.19]	[-0.03; 0.14]
Other endorsements	-0.27	-0.14	-0.23
	[-0.34; -0.19]	[-0.22; -0.07]	[-0.28; -0.17]
Vaccine attitude	0.10	0.22	0.13
	[-0.06; 0.26]	[0.10; 0.34]	[0.05; 0.21]
Gender, male	-0.36	-0.21	-0.44
	[-0.63; -0.08]	[-0.43; 0.00]	[-0.59; -0.29]
Education, university	-0.02	0.09	-0.28
	[-0.28; 0.23]	[-0.17; 0.35]	[-0.44; -0.14]
Age	0.00		0.01
	[-0.01; 0.01]		[0.00; 0.01]
Age, less than 30		-0.20	
		[-0.44; 0.04]	
Age, more than 60		-0.06	
		[-0.30; 0.19]	
Age, no answer		4.47	
		[4.18; 4.77]	
Ideology, DKNO		-0.06	0.05
		[-0.48; 0.38]	[-0.51; 0.63]
Ideology	0.07	-0.49	0.81
	[-0.45; 0.60]	[-1.11; 0.13]	[0.54; 1.07]
Cut point 1	-1.33	-1.23	-0.76
	[-1.90; -0.79]	[-1.67; -0.78]	[-1.04; -0.49]
Cut point 2	-0.82	-0.80	-0.27
	[-1.38; -0.27]	[-1.23; -0.38]	[-0.54; 0.02]
Participants	832	1,474	1,001
Observations	8,320	14,740	10,010
