# Supplementary figures and images for: Assessing WHO’s influence: A randomized conjoint experiment on vaccine endorsements in diversified global health systems
Source: PLOS Glob Public Health. 2025 Nov 21;5(11):e0005410. doi: 10.1371/journal.pgph.0005410 (PMC12637889; doi:10.1371/journal.pgph.0005410)

## Canada

## Japan

## USA

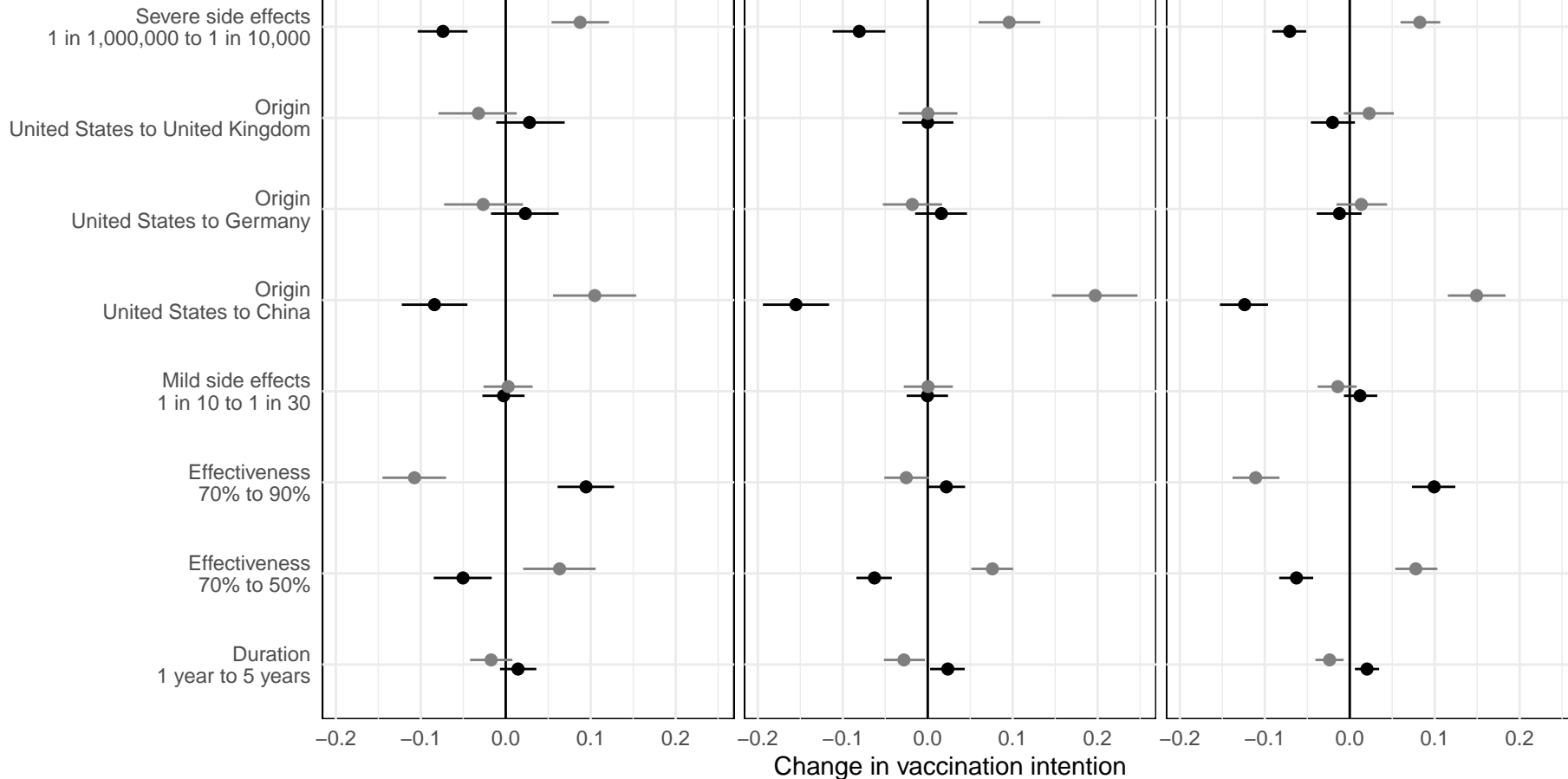

Taking when?

Early

Late

Supplement: S2 Fig — This figure gives the effects of changes to the efficacy and safety profile as well as to the country of origin. The black dots and lines give the changes to the probability to taking the vaccine late, and the gray counterparts to the probability of taking it swiftly. Lines are 95% confidence intervals. (PDF) [file pgph.0005410.s008.pdf]
